# Nutrients Recovery from Dairy Wastewater by Struvite Precipitation Combined with Ammonium Sorption on Clinoptilolite

**DOI:** 10.3390/ma14195822

**Published:** 2021-10-05

**Authors:** Claver Numviyimana, Jolanta Warchoł, Bartosz Ligas, Katarzyna Chojnacka

**Affiliations:** Department of Advanced Material Technology, Faculty of Chemistry, Wroclaw University of Science and Technology, 50-372 Wroclaw, Poland; jolanta.warchol@pwr.edu.pl (J.W.); bartosz.ligas@pwr.edu.pl (B.L.); katarzyna.chojnacka@pwr.edu.pl (K.C.)

**Keywords:** phosphorus recovery, dairy wastes, struvite precipitation, clinoptilolite, ammonium sorption, spontaneity

## Abstract

Struvite precipitation from Wastewater involves an excess of ammonium to create a supersaturated initial solution. The remaining fraction can be a threat to the environment. This work combined struvite precipitation and ammonium sorption using natural zeolite to decrease the ammonium level in the effluent. Two approaches of estimation of feed sample doses were used. One consisted of gradient experiments for ammonium precipitation to the asymptotic level and was combined with clinoptilolite to lower the ammonium level in the effluent. This approach used doses of 0.05:1.51:0.61:1 of Ca:Mg:NH_4_^+^:PO_4_^3−^ mole ratios, respectively. In contrast, three level design with narrowed NH_4_^+^:PO_4_^3−^ range reached 0.25:1.51:0.8:1 for Ca:Mg:NH_4_^+^:PO_4_^3−^ mole ratios. The addition of zeolite decreased effluent ammonium concentration. In both ways, the P and N recoveries were higher than 94% and 72%, respectively. The complexity of the precipitation mixture decreased the ammonium sorption capacity (Q_e_) of clinoptilolite from Q_e_ of 0.52 to 0.10 meq∙g^−1^ in single and complex solutions, respectively. Thermodynamically, the addition of 1.5 % of clinoptilolite changed the struvite precipitation spontaneity from ∆*G* of −5.87 to −5.42 kJ·mol^−1^ and from 9.66 to 9.56 kJ·mol^−1^ for gradient and three level experimental procedures, respectively. Thus, clinoptilolite demonstrated a positive effect on the struvite precipitation process and its environmental impact.

## 1. Introduction

Struvite is a phosphate salt formed by the association reaction of magnesium, ammonium and phosphate in aqueous alkaline conditions [1]. The lower solubility property and the provision of both nitrogen and phosphorus (P) makes struvite a commercially valuable product for agriculture [2]. The plant nutrients management recommends the use of slow nutrient release fertilizer among which struvite has been qualified by several studies [3,4,5]. Inorganic fertilizers are criticized for high nutrients leaching, which poses environmental pollution of water and eutrophication caused by P flux from agricultural activities [6]. As mitigation, bio-based fertilizers and slow nutrients release-phosphate salts are recommended. Calcium and magnesium phosphate salts such as hydroxyapatite and struvite demonstrated the low nutrient leaching properties, even in sandy soils [7]. Along with slow nutrient release fertilizer awareness in agriculture, the P recovery from industrial effluents is an important area. The European Innovation Partnership Agricultural Productivity and Sustainability (EIP-AGRI) highlighted chemical precipitation for P recovery from wastes [8]. Calcium phosphate was reported to be recovered from manure, while struvite is the recommended form for fertilizer use [8]. The latter is more profitable due to its content of magnesium, nitrogen and P in the forms efficiently available to plants [9,10].

The production of struvite involves an equimolar reaction of magnesium, ammonium and phosphate in aqueous solution. The P recovery from complex aqueous matrices involves higher dose of magnesium and ammonium to create a supersaturated mixture enhancing precipitation and competition with other ions. The latter include metal ions such as aluminum(III), calcium(III) and iron(II/III). The phosphates formed with these ions precipitate better than struvite. Therefore, the optimum conditions for struvite precipitation were experimentally proved to require the excess of precursors in the feed reactor, particularly the NH_4_^+^:PO_4_^3−^ molar ratio. This was studied by several researchers on various Wastewaters. The reported molar ratios NH_4_^+^:PO_4_^3−^ vary from 1:1 to 1:20 [11] to recover phosphorus from complex wastes by struvite precipitation. The excessive ammonium becomes particularly compulsory in struvite precipitation from dairy wastes and other high calcium and multivalent cations containing matrices. Whilst the quality of recovered product is enhanced, the process life cycle assessment (LCA) reports a re-pollution caused by residual ammonium [12]. In fact, from the initial supply in the reactor, only a fraction is involved in struvite formation, while the remaining portion becomes an environmental pollutant. Gong et al. demonstrated that an NH_4_^+^:PO_4_^3−^ mole ratio less than 1.2 in struvite crystallizer enhances ammonium recovery, while less than 65% of ammonium was recovered when NH_4_^+^ moles increased to more than double those of phosphate [13]. Despite that, several reports indicated the need for a higher NH_4_^+^:PO_4_^3−^ molar ratio of substrate during P recovery by struvite precipitation. These include the reported NH_4_^+^:PO_4_^3−^ mole ratio of 3.1:1 applied on swine Wastewater [14]; 5:1 on Wastewater sludge [15]; 2.69:1 on whey [16], 6:1 on municipal Wastewater [17]; and 2.25:1 from industrial Wastewater [18]. Thus, there is a reasonable expectation of ammonium residue from crystallization effluent. Given that P was classified as a non-renewable resource, its recovery to a commercial product is important. However, the improvement of the process nexus life cycle, particularly the environmental impact of residual ammonium, is still a gap and needs to be addressed. The latter is standardized to a threshold of 60 mg∙L^−1^ of ammonium nitrogen in effluent, while P is up to 2 mg∙L^−1^ [19].

The International Organization for Standardization (ISO) has established a methodology for conducting LCAs, which, besides the definition of a goal and scope, inventory analysis and interpretation of results, includes process impact assessment [20]. An unclosed loop for ammonium removal in subsequence of P recovery has negative impacts on the environment such as the increase of eutrophication and the non-conformity to standards of process life cycle [12,20,21]. In this regard, the study combining struvite precipitation with ammonium removal is important for green process implementation. Different approaches were previously reported, including ammonium removal using packed beds with synthetic and natural zeolitic minerals [22]. The most widespread representative of this crystalline, microporous aluminosilicates group is clinoptilolite. This material has a high affinity for ammonium ions and enables their efficient removal from Wastewater. The resulted NH_4_^+^ saturated clinoptilolite can be applied as a fertilizer in agriculture. It is beneficial for both soil by improving water retention and for crops by increasing the N availability (reduction of nutrient losses due to leaching, runoff and volatilization) [23]. The struvite crystallization combined with NH_4_^+^ adsorption on natural zeolite has been proposed as a method for nutrient recovery from human urine [24] and swine Wastewater [25].

The current work aims to simultaneously elaborate methods of obtaining two fertilizer formulations during a single process that combines struvite precipitation from dairy Wastewater and ammonium sorption on clinoptilolite. Moreover, the effect of zeolite addition was determined from a thermodynamic point of view at struvite precipitation equilibrium. The research scope intends to contribute to the environmentally friendly process of P recovery through struvite precipitation.

## 2. Materials and Methods

### 2.1. Chemicals, Reagents and Basic Equipment

The Magnesium chloride, di-potassium hydrogen phosphate, calcium chloride di-hydrate, sodium hydroxide and ammonium chloride were used as magnesium, phosphorus, calcium and alkali source, respectively. All reagents with a purity of 99.5% were obtained from Avantor Performance Materials Poland S.A. Millipore Simplicity UV (Merck, Germany), a laboratory demineralization system that was used to create deionized water. For the latter analysis, ethylic alcohol, nitric acid (65%), hydrogen peroxide (30%) and sulphuric acid (>95%) (Avantor Performance Materials Poland S.A., Gliwice, PL) were used in cleaning materials. In ICP-OES analysis, a multi-element standard (Sigma-Aldrich Chemie GmbH, Taufkirchen, Germany ) was employed for standard calibration. The nitrogen (N) and carbon (C) total contents were determined using the CN analyzer (Vario MACRO Cube elemental analyzer with a Thermal Conductivity Detector—TCD ELEMENTAR Analyser system GmbH, Langenselbold, Germany)). Boric acid, mixed indicator of green bromocresol and methyl red, and hydrochloric acid analytical weight for 1 L 0.1 N were used in total nitrogen digestion, ammonium collection and titration, respectively. The sample of zeolite rock used in this work was collected from a mineral deposit located in the Nižný Hrabovec, Slovakia. The material consists of 74% clinoptilolite, 11% cristobalite, 6% plagioclase, 4% illite and smectite, 3% tridymite, 1% kaolinite and 1% quartz. They are characterized by a specific surface area of 29 m^2^·g^−1^ [26] and a cation exchange capacity of 121–125 meqNH_4_^+^ (100 g)^−1^ [27]. The chemical composition of the zeolitic material was determined through X-ray fluorescence (XRF) analysis.

### 2.2. Sample Preparation

An artificial solution was prepared with the elemental composition corresponding to cheese production Wastewater. The locally characterized whey from Sery Lutomierskie (Poland) cheese producing cooperative contained 512 ± 25 mg·kg^−1^ of P. Heavy metals and other microelements such as Ni, V, Co, Ba, Pb, Sb, Ti, Mn, Fe and Al were in traces (<2 mg·kg^−1^). The artificial sample was prepared following initial conditions with the proper molar ratios favoring struvite precipitation, as previously determined [16]. Those conditions were 0.26, 1.21 and 2.69 for Ca:P, Mg:P and NH_4_^+^:P, respectively. The composition of the sample and initial artificial mixture is presented in Table 1.

### 2.3. Preparation of Zeolite Material

The natural zeolite was characterized for sorption properties, as per referred work [28]. Prior to all experiments, the zeolite was sieved to a granulometric size of 0.16–0.25 mm and pre-treated to obtain homoionic Mg-form by three cycles of contact with 3% MgCl_2_ solution overnight, followed by washing with deionized water and then drying at 105 °C, referring to existing works [29].

### 2.4. Ammonium Sorption Experiment

The ion exchange of NH_4_^+^ on Mg-clinoptilolite was examined in batch mode experiments. A measured quantity of mineral (1 g) was added to 50 mL vials containing a measured volume of NH_4_^+^ solution (40 mL) in the range of 0–500 mg·L^−1^ for a contact time of 24 h to reach the sorption equilibrium. Subsequently, samples were decanted and filtered by using Whatman nylon filters with 0.45 μm of pore size. The EPA method 350.2 was used to determine the residue ammonium content. The amount (*q_e_*) adsorbed was calculated in terms of initial (*C*_0_) and equilibrium (*C_e_*) NH_4_^+^ concentrations, mass of mineral (*m_z_*) and NH_4_^+^ solution volume (*V*), as described by the following equation (Equation (1)).

(1) $$q_{e}=\frac{\left(C_{0}-C_{e}\right)*V}{m_{z}}$$

For this study, the single ion solution of ammonium ions in a concentration range of 0–500 mg·L^−1^ was used. In order to evaluate the effect of other ions on ammonium sorption, the same equilibrium experiments were conducted on multi-components aqueous solution residue from struvite precipitation with the same range of ammonium concentration. The sorption experiments were additionally done on both raw and Mg-form of zeolitic material to assess their effect on ammonium uptake.

### 2.5. Struvite Precipitation Experiments

The adjustment of pH was done using aqueous sodium hydroxide solution (6 M NaOH) and hydrochloric acid (1 M HCl) to exact pH 8.9 ± 0.1. The molar ratio of calcium, magnesium and ammonium to P were adjusted in initial batch reactors by adding the pre-calculated quantity of their solution to 100 g of mixture. In this case, ammonium chloride (4 mol·kg^−1^), dipotassium hydrogen phosphate (1 mol·kg^−1^), calcium chloride (1 mol·kg^−1^) and magnesium chloride (2 mol·kg^−1^) were used. The effluent was characterized for phosphorus, magnesium and ammonium concentrations. The stirring rate of 100 rpm, a reaction time of 60 min and 1h for liquid-solid phase equilibrium were set, as described in other works [18,30]. The dry solid precipitate with and without zeolite were characterized using X-ray diffraction (XRD) (Rigaku MiniFlex diffractometer, Tokyo, Japan) and scanning electron microscopy-Energy dispersive spectroscopy (SEM-EDS, the SEM/Xe-PFIB Microscope FEI Helios PFIB from Massachusetts, United States) as in previous studies [16]. The nutrients’ composition in the products was analyzed for the total concentration of elements using a standard method of multi-elements analysis (USEPA method 3051) [31]. The evaluation of products as fertilizer was assessed by a germination test and nutrient dissolution in citric acid 2% adjusted at pH 6 with NaOH 6N [32,33].

### 2.6. Effect of Zeolite on Precipitation Process

In addition to the effect on the final effluent ammonium concentration, the evaluation of the zeolite effect on struvite precipitation was based on thermodynamic parameters affecting the equilibrium. These are enclosed in the Gibbs energy of the precipitation spontaneity described by Equation (2) [34]

(2) $$∆G=-2.303\frac{RT}{n}\log\left(\frac{IAP}{Ksp}\right)$$

where ∆G is the the Gibbs free energy in KJ·mol^−1^ for precipitation reaction, *R* is the ideal gas constant (*R* = 8.31447 × 10^−3^ KJ·mol^−1^·K^−1^), T is the absolute temperature (295 K), *n* is the number of crystal lattice ions, the ion activity product (IAP), which is a conditional solubility product ($IAP=\overset{n}{\underset{i=1}{\prod }}a_{i}$), *K_sp_* is the solubility product and *a_i_* is the activity of lattice ion standing for $a_{NH_{4}^{+}}$, $a_{Mg^{2+}}$, $a_{PO_{4}^{3-}}$. for struvite. The activities are obtained from the concentration of each component corrected with activity coefficient in dependence of solution ionic strength. The equilibrium is reached when ∆G=0, spontaneous for precipitation when ∆G<0 and under-saturation when ∆G>0. Thus, the discussion of the effect of zeolite on the process condition was described as the extent to which struvite thermodynamic equilibrium is reached. Considering the complexity of the feed sample, the molar balance of each lattice ion component was estimated to find the activity obtained as dependent on the fraction ($\alpha_{i}$) of the total activity ($a_{T}$. ) of the component ($a_{i}=\alpha_{i}*a_{T})$. The speciation analysis was done using Visual MINTEQ (version 3.1, KTH, Sweden). For the status of equilibrium, the total quantity of a component in the influent was preliminarily estimated to reach the minimum of P and ammonium. This considers both the suitable concentration range recommended for the evaluation of ammonium sorption by natural zeolite (<500 mg·kg^−1^) [28] and the reported high ammonium concentration in struvite precipitation effluents, as per previous studies [13]. Therefore, the precipitation equilibrium experiments consisted of the adjustment of the competing ions to 0.26 Ca:P molar ratio and the reaction conditions of pH 8.9 [16]; other parameters were assessed towards the removal of both P and ammonium. Two approaches were used to find the feed material dose for coupling with sorbent material for the evaluation of their effect on equilibrium. These include:

#### 2.6.1. Gradient Batch Experiments

The high ammonium concentration in struvite reactor effluent underwent further batch gradient precipitation with the aim of ammonium removal to the minimum. In this case, the gradient descent experiments were conducted iteratively in the batch reactor [35]. The objective was to find the total feed material of struvite lattice components that precipitates ammonium until the minimum concentration is reached [36]. This was described by a relation of concentration ($C_{NH_{4}^{+}}$) in the function of the monotonous repeated batch experiments standing for the design variable (*x*). Up to five descent experiments were necessary to reach the asymptotic level. The volume was upgraded to initial batch, and the dilution factors were involved in subsequent experiment calculations to keep the volume constant. In this case, the variation of ammonium concentration decreased from the initial value *C*_0_ to *C_f_*. An integrated relation of that decrease in function of gradient experiments is presented in Equation (3) and was used to fit the obtained data, as described by [36]. In Equation (3), Chua et al. (2012) evaluated ammonium removal in a function of time up to the minimum final concentration (*C_f_*). In this work, the number of iterated gradient experiments was instead considered.

(3) $$C_{NH_{4}^{+}}=\left(C_{0}-C_{f}\right)e^{-kx}+C_{f}$$

Furthermore, the estimated minimum is influenced by the initial supersaturation of struvite. Thus, the investigation was done on three different gradients considering Mg: P molar ratios. These include molar ratios Mg:P 1.21; 1.51; 1.81, which enhance the various range of initial concentration of lattice ions and struvite supersaturation. The Equation (3) was fitted with the experimental results that reached the smaller experimental value of $C_{NH_{4}^{+}}$ after five sequential repetitions. The optimization problem was defined mathematically as looking for $\left(\min C_{NH_{4}^{+}}\left(x\right)\right)$, with the aim of finding an iteration batch number needed to minimize the concentration using descent methods [37]. The optimum conditions are achieved when the slope of obtained function becomes zero, i.e., $df\cdot dx^{-1}=0$. Given that Equation (3) is continuous, the tolerance of 10^−3^ was set during $df\cdot dx^{-1}$ iteration. The calculated sum of molar flow to batch number x^*^ was applied in the batch reactor where the combination with 1.5 percent of zeolite served to evaluate their effect on struvite precipitation and ammonium sorption.

#### 2.6.2. Combining Sorption and Desirability Approach

The approach consisted of further investigation of Mg:P and narrowed NH_4_^+^:PO_4_^3−^ ratios suitable for use after the molar adjustment of potential competitive ions with a proper additional P dose. Under this three-level experimental design, the range of the investigated NH_4_^+^:PO_4_^3−^ ratio was 0.15–1.45, while Mg: P ranged from 1–2. In addition to P recovery (RecP) and struvite precipitation (X-NH_4_^+^), the ammonium recovery (RecN) was included in the output. The data were analyzed to meet the target of each output. The obtained values of factors with good desirability were used for the single batch reactor with and without addition of zeolite.

### 2.7. Germination Tests

The germination tests were conducted during 14 days on cucumber (*Cornichon de Paris*). The seeds were cultivated on humic soil that contained no fertilizer. The struvite fertilizer was added considering the ratio of 3 g struvite per 500 g soil, as found in other works [38]. The cultivated seeds were illuminated with a specific lamp (LED Secret Jardin COSMORROW 20 W) and watered daily after 24 h. The germination rate and biomass yield were used for the interpretation of the fertilizer efficiency. The harvested biomass was dried and analyzed for elemental composition to assess nutrients’ uptake by plant. The Transfer Factor (TF) was determined using Equation (4):

(4) $$TF=\frac{C_{x}\cdot m_{p}}{C_{f}\cdot m_{f}}$$

where *TF* is the transfer factor, *C_x_* is the content of the component in the dry weight of the sprout (mg·kg^−1^), *m_p_* is the mass of the plant (kg), *C_F_* is the content of the component in the fertilizer (mg·kg^−1^) and *m_f_* is the mass of applied fertilizer (kg). The roots analysis was conducted on a WinRHIZO Regular STD400 apparatus (Instrument Regents, Québec, QC, Canada) according to the validated procedures [39].

## 3. Results

### 3.1. Characteristics of Zeolite Material

**Figure 1:**
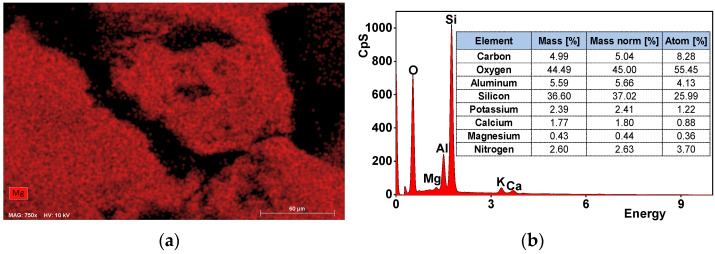

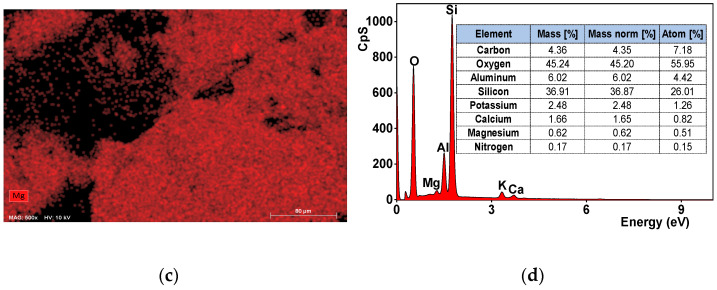
SEM image of zeolite with magnesium mapping (**a**) and EDS (**b**) spectra, and their respective image (**c**) and spectra (**d**) for Mg-form activated zeolite.

The XRF results of row, modified and ammonium contacted forms of zeolites are presented in Table 2. The obtained XRF results are in agreement with other works in regard to Al and Si as the main components of the zeolitic tuff [40]. The Mg^2+^ sorption occurs via ion exchange reaction mainly onto the internal porosity of zeolite and on the external surface in the minority. The SEM-EDS (Figure 1a–d), at randomly chosen points on the external surface, confirms lower magnesium content on non-treated zeolite than on its Mg-form.

### 3.2. Sorption and Precipitation Results

**Figure 2:**
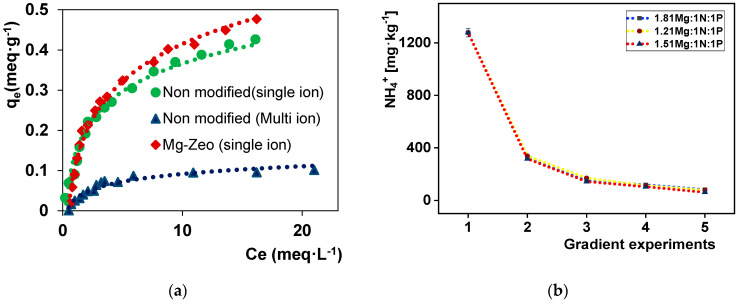
(**a**) Ammonium sorption equilibrium results; (**b**) gradient ammonium removal.

The ammonium sorption characterization results obtained by the equilibrium experiment for the natural zeolites and their modified form are presented on Figure 2a. The values of *q*_m_ for ammonium uptake were 0.52 and 0.67 meq·g^−1^ for natural and modified sorbent material, respectively. Compared to non-activated zeolite, their Mg-form demonstrated higher ammonium sorption capacity. In real solution with a high concentration of monovalent ions such as sodium, and potassium using struvite precipitation effluent, the ammonium sorption decreased up to 0.1 meq·g^−1^, which highlights the effects of other cations on sorbent active sites. The comparison of those aspects is shown on Figure 2a.

For ammonium removal by precipitation, the gradient experiments in batch reactions conducted at the same pH 8.9 with molar ratios of 1.21Mg:1N:1P, 1.51Mg:1N:1P and 1.81Mg:1N:1P are presented in Figure 2b. The experiments run up to five batches indicate the ammonium decrease in every batch in descending manner. In comparison with other investigated molar ratios for ammonium precipitation, the 1.51Mg:1N:1P gradient reached the smallest ammonium concentration. This was chosen to find the relation by which an iteration batch number significantly minimizing the concentration was estimated.

The obtained relation equation was y = 80.5 + 5908 exp(−1.60*x*) and the corresponding x* = 11 with a tolerance value *ϵ* = *f*′(x*) = 3 × 10^−4^. The latter was less than the set tolerance limit (10^−3^). The data were used to estimate the total feed molar flows of dosage needed to remove ammonium by struvite precipitation to the minimum concentration level. The total dosage thereby obtained was applied in the reactor to 1 kg artificial and real Wastewater, which indicated a P and ammonium recovery of 99.05% and 95.49%, respectively. The effluent concentrations at equilibrium (Table 3) decreased from 444.79 to 4.21 mg·kg^−1^ and 1869 to 84.60 ± 7.63 mg·kg^−1^ for P and ammonium, respectively. The addition of zeolite up to 1.5% of the reaction volume improved the ammonium recovery to 97.44%. The applied dose from the gradient descent optimization supplemented with zeolite enhanced the equilibrium ammonium decrease from 84.4 to 48.7 ± 7.63 mg·kg^−1^, while P kept a significantly low value of 4.23 mg·kg^−1^, thus underlining the sorption of ammonium by natural zeolite.

**Figure 3:**
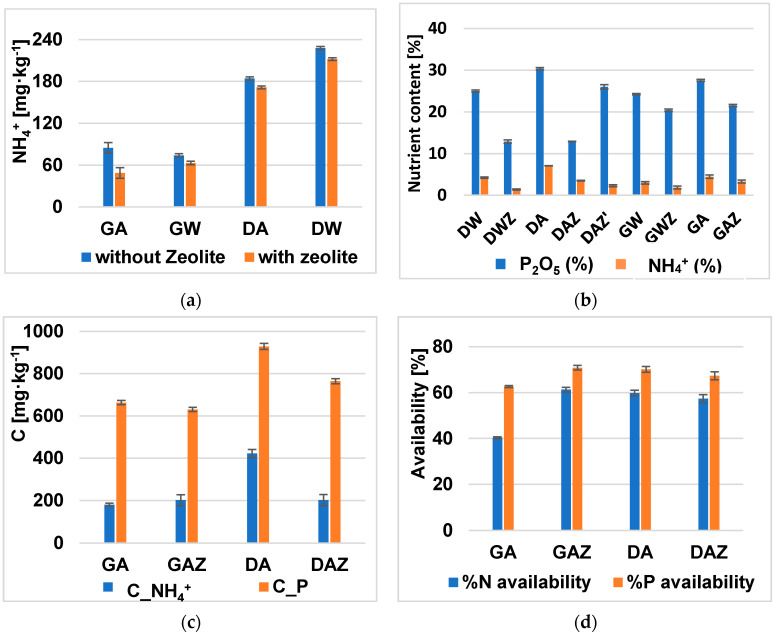
Comparison of ammonium concentration in effluents obtained from (**a**) GA: batch applied from gradient with artificial solution (GA) and real Wastewater sample (GW); batch applied from desirability approach with artificial (DA) and real Wastewater (DW); (**b**) Nitrogen and phosphorus content in their experimental products without and with zeolite (GAZ, GWZ, DAZ, DWZ); their nutrient availability in citric acid as concentration (**c**) after 2 h; and as % (**d**) of dissolved product.

On the other side, the combination of the desirability approach and sorption with the prior adjustment of struvite inhibitors (calcium) has achieved a dosage of 1.51:0.8:1 of Mg:NH_4_^+^:PO_4_^3−^ with a desirability of 0.77. The ammonium and P recoveries, and ammonium content in struvite precipitate were 72, 99 and 6.02%, respectively. The addition of zeolite enhanced ammonium removal, lowering its content from 184 to 174 mg·kg^−1^ in the effluent. The advantage of this combination is the use of a smaller quantity of the influent ammonium capable of favoring the product nitrogen content. Nevertheless, the effluent ammonium concentration was higher than in gradient experiments due to initial higher Ca: P mole ratio, thus affecting struvite precipitation. This was observed in both artificial sample solution (DA, DAZ, and DAZ) and waste samples (DW and DWZ). Nevertheless, the addition of zeolite decreases the fraction of NH_4_^+^ and P in the products. As a mitigation, the addition of zeolite was decreased from 1.5 to 0.75 percent for samples DAZ and DAZ’, respectively. The comparison of ammonium removal is presented in Figure 3a, while the experimental data on nutrients content are compared in Figure 3b. The additional experimental data are presented in Table A1 and Table A2 of the Appendix A and Appendix B, respectively. The recoveries and effluent concentrations are presented in Table 3.

The significance of zeolite effect in ammonium removal is attributed to its sorption properties to cations. In addition, these materials provide the surface area for reaction, thus playing a role in crystal growth by lattice ions colliding on their surface area [41]. Considering the obtained removal efficiency in the batch mixture, the ammonium and P are efficiently recovered and struvite produced using smaller molar ratios of NH_4_^+^:PO_4_^3−^, and Ca: P. The Mg: P, therefore, had to be higher to enhance struvite supersaturation.

Upon gradient descent experimental investigation of ammonium precipitation, the total feed mass balance was found with 0.05Ca: 1.51Mg:0.61 NH_4_^+^:1PO_4_^3−^. Under these initial conditions, ammonium and P were efficiently removed, rendering the process nexus lifecycle and increasing productivity due to combined P and ammonium recovery. In the counter approach of desirability with sorption process, the molar ratios were found to 0.26Ca:1.51Mg:0.8 NH_4_^+^:1PO_4_^3−^. The lower NH_4_^+^:PO_4_^3−^ molar ratio have been used previously and enhanced both P and ammonium recovery by struvite precipitation [13]. In this case, the necessity of a greater amount of magnesium becomes compulsory to maintain the thermodynamic equilibrium (IAP) of struvite and to achieve more ammonium removal. This approach involved a smaller quantity of added salts. However, due to a higher Ca:P mole ratio of 0.26, the ammonium in effluent was higher than for gradient experiments, which used a quite lower Ca:P mole ratio.

### 3.3. Thermodynamic Aspect

The zeolite effect on equilibrium was assessed based on supersaturation ratio, with the accountability of struvite precursors species at equilibrium (Table 4). The speciation indicated different component species such as Mg^2+^, MgCl^+^ and MgOH^+^, which were used to constitute the active molar fraction of magnesium [42]; the NH_4_^+^ and NH_3_ were used to constitute the ammonium molar fraction [43], while PO_4_^3−^, HPO_4_^2−^ and H_2_PO_4_^−^ were used to find the molar fraction of orthophosphate as struvite precursors. The IAP and the ∆G. demonstrated the difference for experiments upon zeolite addition. There is a slight change of ∆G, which is due to the quantitative expressions involved in the estimation of thermodynamic parameters. In fact, the effect encountered by zeolite addition enhances the change of struvite spontaneity up to −0.4 and −0.11 KJ·mol^−1^ for the effluents of products GA and GAZ and DA and DAZ, respectively.

The ∆G. in Table 4 highlights the enhanced trend to equilibrium energy (∆G=0) due to the addition of zeolite. The positive effect is found on the final ammonium concentration in the effluent, as described in Section 3.2. This is an advantage in the mitigation of the environment pollution caused by the excessive ammonium in struvite precipitation effluent.

### 3.4. Characterization of Recovered P Products

#### 3.4.1. XRD and SEM Results

**Figure 4:**
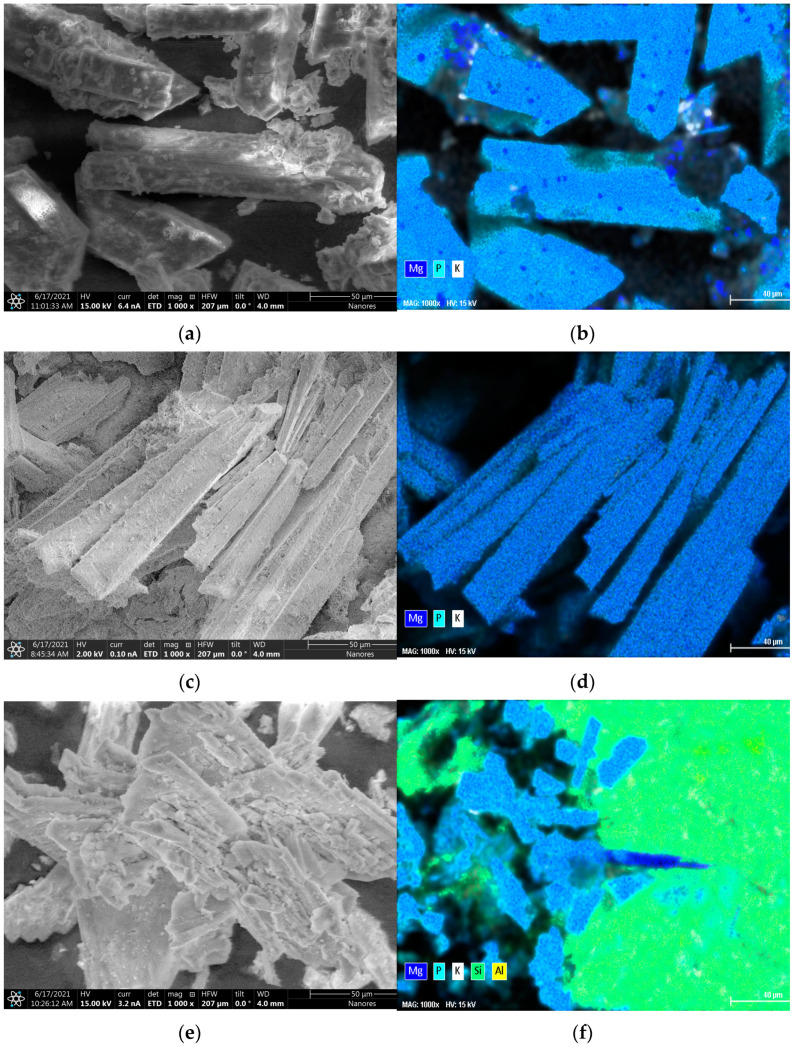
SEM-EDS images for DA (**a**) and its elemental mapping (**b**); for GA (**c**) and its elemental mapping (**d**); for GAZ (**e**) and its elemental mapping (**f**).

The XRD and microscopic characterization of the precipitates indicates the high crystallinity of the material. The orthorhombic structures in Figure 4 viewed in SEM are struvite witness [44], thus confirming the P recovery product with high struvite content.

**Figure 5:**
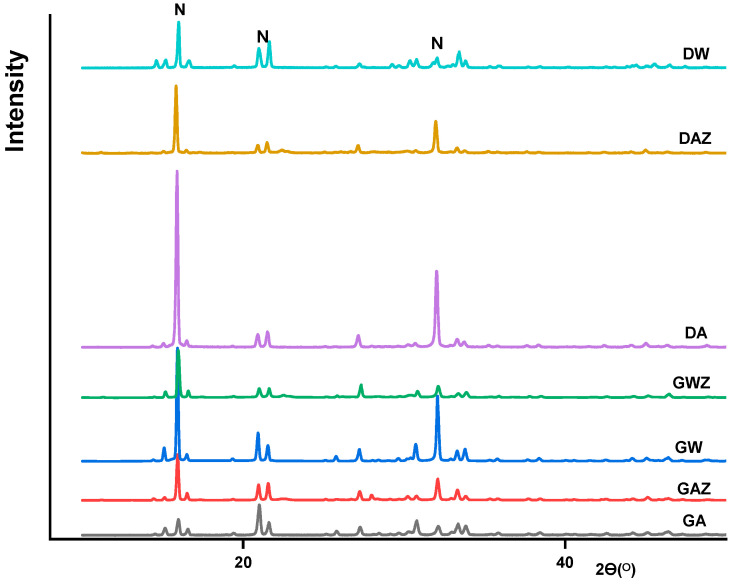
XRD spectra for recovered products from artificial solutions with and without zeolite, respectively.

Furthermore, due to the excessive use of K_2_HPO_4_ in the reactor feedstock, the K-struvite is predominant in all products of gradient experiments, while N-struvite is formed at a lower extent. In fact, both forms exhibit closer lattice parameters that are hard to distinguish. In the characterized products, the crystal lattice parameters of 31 Pmn21 space group were observed for all products. Small differences were found on the lattice parameters a: 6.955, b: 6.142 and c: 11.218 A^ᴼ^ for N-struvite crystals, while they were a: 6.892, b: 6.166 and c: 11.137 A^ᴼ^ for K-struvite. That makes their peaks overlay in the XRD spectra. The two forms have their characteristic peaks at 15.9, 20.8, 21.5 and 31–32 Ɵ. The XRD spectra of all products are presented in Figure 5. In correlation with the Table 3, the diffraction peak intensity at 15.9 Ɵ increases with the nitrogen and magnesium content accompanied by a potassium decrease in the product. The obtained descriptive properties of the products are in agreement with previous crystalline studies for struvite [45]. The intensity decreases with the matrix effect as well as with the used method. The products of gradient precipitation have a lower concentration of nitrogen and an increased concentration of potassium; thus, K-struvite is predominant in those products. In fact, this is due to the high amount of K_2_HPO_4_ used in the reactor. In contrast, the products with influent components estimated by the desirability approach have a high nitrogen content, highlighting a better formation of N-struvite. Moreover, in contrast to experiments without zeolite, the crystals are more agglomerated on the zeolite material surface (Figure 4e,f). This is enhanced by the area provided during crystal growth as well as the interaction between struvite crystal lattice components with an active site of the magnesium homo-ionic form of the sorbent materials.

#### 3.4.2. Evaluation of Fertilizer Properties

In both production processes, the obtained material is rich in phosphorus (up to 30 percent of P_2_O_5_), nitrogen and potassium (Figure 3c). Furthermore, all of the product nutrient release assessed in citric acid was effective in releasing nutrients in an efficient manner (Figure 3c,d). Additionally, the germination tests for DA and DAZ were successful. The seeds germinated during two weeks, with an 88, 76 and 72% germination rate for the trial fertilized with struvite, struvite with zeolite and control without fertilizer, respectively.

The results from the biomass of cucumber (Table 5) demonstrates that the application of the new struvite-zeolite based fertilizer can significantly improve the nutrient content in the harvest. Compared to the control test, macronutrients were more concentrated in the biomass of fertilized trials. This difference highlights the quality of struvite to serve as a source of plant nutrients. The magnesium uptake was nearly a thousand times higher in fertilized trials than in control, which indicates the struvite benefits for crops. This effect was previously underlined by other studies, where Mg and K play a potential role in the regulation of cation-anion imbalance in plant cells [46].

The nutrients’ TF was also determined for fertilized treatments. The transfer of elements to cucumber biomass depended on the chemical form of the nutrient supplied in the fertilizer. In the case of potassium (K), the highest TF was achieved for the DA. In case of other elements, the struvite combined with zeolite has demonstrated a better TF. Moreover, the highest fresh sprout mass (88.70 g) was achieved in the DAZ group. The obtained sprout mass was about 96% higher compared to the DA (Table 6). For all considered growth parameters, significant differences were observed between almost all groups. Similar results were reported in other struvite application experiments [47,48].

Considering the biomass study presented in Table 6, an inclusion of zeolitic material to the soil improved the roots development of cucumber. It results from the induction of aquaporin structure, which enhances water uptake and contributes to an increased amount of fresh biomass harvested for trial fertilized by struvite combined with zeolite [49].

## 4. Conclusions

The improved struvite precipitation process identified in this work highlights the use of smaller N:P and Ca:P mole ratios to avoid an excess of ammonium in the effluents and struvite inhibition, respectively. The process effluent ammonium concentration was increased from 84 to 184 mg·kg^−1^ when the feed molar ratio changed from 1.51Mg:0.61 NH_4_^+^:0.05Ca:1P to 1.51Mg:0.8NH_4_^+^:0.26Ca:1P, respectively. In both cases, the ammonium removal efficiency was significantly improved by the combination with natural zeolite up to 1.5 percent of the feed sample mass. Considering the economic aspect of the process, the smaller N:P feed mole ratio combined with the sorption process required a smaller amount of feed material in the reactor to efficiently recover P and ammonium with high struvite content in the final product. The initially promising fertilizer properties were shown in germination tests. The new formulation of struvite-zeolite demonstrated a significant contribution to the plant mass. The obtained results can find practical application as an environmentally friendly and economic method of struvite precipitation with both nitrogen and phosphorus recovery.

## Figures and Tables

**Table 1:** Initial concentration of the artificial sample and its corresponding concentration in real Wastewater.

Concentration [mg∙kg^−1^]					
Elements	Wastewater	Artificial	Elements	Wastewater	Artificial
pH	4.35	8.9	N [%]	0.14	-
C [%]	3.07	-	Na	485	-
Cl^−^ [mol·kg^−1^]		0.16	P	445	1203
B	10.3	-	S	132	-
Ca	526	404	Si	99.94	-
K	1654	4524	NH_4_^+^	364	1876

**Table 2:** XRF characteristic results for activated (z-Mg), raw zeolites and after ammonium contact.

	z-Mg	Raw	after NH_4_^+^ Contact
Compound	m/m%	m/m%	m/m%
SiO_2_	64.22	68.30	59.53
Al_2_O_3_	8.39	14.28	8.01
K_2_O	8.33	4.77	11.45
CaO	7.65	6.33	9.94
Fe_2_O_3_	4.96	2.70	4.90

**Table 3:** P and other element concentrations in effluent, and the content of nutrients in the struvite-zeolite fertilizer.

	Effluent Concentration				Product Nutrient Content				
	[mg·kg^−1^]				%				
Sample	Ca	Mg	P	NH_4_^+^	N	P_2_O_5_	K	Ca	Mg
GA	45.2	1322	4.21	84.6 ± 7.63	3.42	29.5	3.05	0.65	12.8
GAZ	62.4	1348	4.32	48.6 ± 7.61	2.02	20.7	2.53	0.85	8.81
GW	78.2	1048	5.81	73.8 ± 2.55	2.21	24.7	1.95	0.25	10.1
GWZ	68.8	1235	5.54	63.0 ± 2.50	1.47	18.1	1.22	0.50	6.38
DA	45.2	282	5.41	184 ± 2.10	5.49	30.3	0.76	4.27	9.80
DAZ	62.4	293	4.80	176 ± 1.70	2.74	26.0	0.32	1.58	10.6
DW	56.7	248	-	228 ± 2.20	3.30	25.0	0.29	2.27	9.76
DWZ	63.5	279	-	212 ± 1.70	1.09	12.9	0.51	1.85	2.94

**Table 4:** Stoichiometric matrix of the struvite components and species molar fractions at equilibrium of different experiments.

Component	Species	Mg^2+^	NH_4_^+^	PO_4_^3−^	Na^+^	K^+^	Ca^2+^	H^+^	CO_3_^2−^	H_2_O	Cl^−^	GA	GAZ α_i_ (%)	DA	DAZ
Mg^2+^	Mg^2+^	1										46.72	50.43	51.53	51.52
	Mg_2_CO_3_^2+^	2							1			5.08	5.32	1.39	1.39
	MgOH^+^									−1		0.05	0.06	0.06	0.06
	MgCl^+^	1									1	14.74	15.87	16.59	16.60
	MgHPO_4_ (aq)	1										0.02	0.05	0.20	0.18
	MgCO_3_ (aq)	1										23.38	19.81	21.23	21.25
	MgHCO_3_^+^	1						1	1			10.01	8.47	8.99	9.00
PO_4_^3−^	PO_4_^3−^			1								0.07	0.06	0.09	0.09
	HPO_4_^2−^			1				1				36.20	31.59	48.06	48.13
	H_2_PO_4_^−^			1				2				0.23	0.20	0.30	0.30
	MgPO_4_^−^			1								1.41	1.52	0.57	0.57
	MgHPO_4_ (aq)	1		1				1				33.56	36.34	13.81	13.84
	CaHPO_4_ (aq)	1		1				1				0.92	2.26	0.81	0.82
	CaPO_4_^−^			1			1					3.40	8.37	2.98	2.98
	NaHPO_4_^−^			1	1			1				18.09	14.46	25.12	25.02
	KHPO_4_^−^			1		1		1				4.68	4.09	6.18	6.19
	K_2_HPO_4_ (aq)			1		2						0.26	0.22	0.34	0.34
	KH_2_PO_4_ (aq)			1		1						0.01	0.01	0.02	0.02
	KPO_4_^2−^			1		1						0.02	0.02	0.02	0.02
	Na_2_HPO_4_ (aq)			1	2							1.06	0.77	1.54	1.53
	Na_2_PO_4_^−^			1	2							0.03	0.02	0.04	0.04
	NaH_2_PO_4_ (aq)			1	1							0.03	0.03	0.05	0.05
	NaPO_4_^2−^			1	1							0.04	0.04	0.06	0.06
NH_4_^+^	NH_4_^+^		1									76.31	76.23	76.12	76.12
	NH_3_ (aq)		1					1				23.65	23.66	23.85	23.85
	CaNH_3_^2+^		1				1					0.04	0.11	0.03	0.03
I (M)												0.36	0.35	0.32	0.32
∆G (Kj.mol^−1^)												−5.87	−5.42	−9.66	−9.56

**Table 5:** The results of nutrient uptake by cucumber fertilized with struvite fertilizer.

Sample	Element Content in Dry Biomass (mg·kg^−1^)					TF				
	N	P	K	Ca	Mg	N	P	K	Ca	Mg
Struvite (DA)	4.42	16.59	48.19	20.71	8547	0.44	0.07	3.47	0.27	0.23
Str & zeolite (DAZ)	3.04	14.98	39.91	19.49	8423	1.04	0.12	1.09	1.58	0.33
Control	2.86	12.52	0.94	14.12	8.61	-	-	-	-	-

**Table 6:** Biomass and roots properties.

Group	Fresh Sprouts Mass	Dry Sprouts Mass	Root Area	Root Length	Steam Length	Root Volume
	[g]	[g]	[cm^2^]	[g]	[g]	[cm^3^]
DA	45.14 ± 4.62	5.62 ± 0.42	8.43 ± 0.34	6.06 ± 1.44	3.03 ± 1.10	0.77 ± 0.12
DAZ	88.7 ± 8.46	8.83 ± 1.11	13.46 ± 1.15	8.32 ± 2.16	2.71 ± 0.73	1.51 ± 0.24
Control	26.2 ± 4.61	4.56 ± 0.43	11.24 ± 0.94	4.66 ± 0.14	1.82 ± 0.46	7.18 ± 0.14

## Data Availability

Not applicable.

## Appendix A

**Table A1:** Gradient Experiments.

Batch n^o^	Mg:P	NH_4_^+^:P	Mg	P	NH_4_^+^
1	1.21	2.69	150	5.819	1278
2	1.81	1.0	311	0.792	320.5
	1.21	1.0	179	2.057	338.7
	1.51	1.0	281	1.479	316.5
3	1.81	1.0	251	5	149.51
	1.21	1.0	108	15	170.27
	1.51	1.0	194	5	143.28
4	1.81	1.0	261	4	118.61
	1.21	1.0	132	66	112.25
	1.51	1.0	231	8	103.78
5	1.81	1.0	281	5	82.60
	1.21	1.0	80	63	78.37
	1.51	1.0	238	12	61.42
f(x)	y = 80.54 + 5908.25exp(−1.597x)				
f’(x)	Y’ = 11453.44exp(−1.597x)				
#iteration	CNH_4_^+^	C’NH_4_^+^	nNH_4_^+^	nP	nMg
			0.104*	0.039*	0.044*
1	1276.455	2318.328	0.071	0.071	0.107
2	322.613	469.260	0.018	0.018	0.027
3	129.543	94.984	0.007	0.007	0.011
4	90.463	19.226	0.005	0.005	0.008
5	82.552	3.892	0.005	0.005	0.007
6	80.951	0.159	0.004	0.004	0.007
7	80.627	0.032	0.004	0.004	0.007
8	80.562	0.007	0.004	0.004	0.007
9	80.548	0.001	0.004	0.004	0.007
10	80.546	0.000	0.004	0.004	0.007
11		3 × 10^−4^			

## Appendix B

**Table A2:** Three Level Design of Experiments.

Run n^o^	Factors		Effluent		Inflow	Outflow		Yields		
	Mg:P	N:P	Eff _(g)_	P_i_	NH_4_^+^_(i)_	P_out_	NH_4_^+^_(out)_	RecN	NH_4_^+^	RecP
								[%]		
1	1.21	0.8	87.5	1252	581.76	7.28	162.68	72.8	4.8	98.6
2	1.81	0.8	90	1252	1054.44	7.06	521.32	70	5.72	99.2
3	1.51	0.8	77.5	1252	109.08	19.48	10.01	68	6.1	99.3
4	1.21	1.45	81.5	1252	1054.44	4.50	815.63	45.1	5.43	99.4
5	1.21	0.15	90	1252	581.76	9.13	157.04	92.4	1.21	97.3
6	1.51	0.15	77.5	1252	109.08	25.81	6.45	92.3	1.25	98
7	1.51	1.45	98	1252	1054.44	5.28	534.85	48.2	7.3	99.6
8	1.81	0.15	77.5	1252	109.08	19.84	6.50	88.2	0.0778	98
9	1.81	1.45	85.5	1252	581.76	14.97	135.50	33.1	7.35	99.6

## References

[1] Lee J.J., Choi C.U., Lee M.J., Chung I.H., Kim D.S. (2004). A study of NH_3_-N and P refixation by struvite formation in hybrid anaerobic reactor. Water Sci. Technol..

[2] Talboys P.J., Heppell J., Roose T., Healey J.R., Jones D.L., Withers P.J.A. (2016). Struvite: A slow-release fertiliser for sustainable phosphorus management?. Plant Soil.

[3] Rahman M., Amran M., Salleh M., Rashid U., Ahsan A., Mujaffar M., Six C. (2014). Production of slow release crystal fertilizer from wastewaters through struvite crystallization—A review. Arab. J. Chem..

[4] Nongqwenga N., Muchaonyerwa P., Hughes J., Odindo A., Bame I. (2017). Possible use of struvite as an alternative phosphate fertilizer. J. Soil Sci. Plant Nutr..

[5] Gómez-Suárez A.D., Nobile C., Faucon M.P., Pourret O., Houben D. (2020). Fertilizer potential of struvite as affected by nitrogen form in the rhizosphere. Sustainability.

[6] Sundareshwar P.V., Morris J.T., Koepfler E.K. (2003). Phosphorus Limitation of Coastal Ecosystem Processes. Science.

[7] Ehmann A., Bach I., Laopeamthong S., Bilbao J., Lewandowski I. (2017). Can Phosphate Salts Recovered from Manure Replace Conventional Phosphate Fertilizer?. Agriculture.

[8] EIP-AGRI EIP-AGRI Focus Group (2017). Nutrient Recycling. https://ec.europa.eu/eip/agriculture/sites/default/files/eip-agri_fg_nutrients_recycling_final_report_2017_en.pdf.

[9] Ballirano P., De Vito C., Mignardi S., Ferrini V. (2013). Phase transitions in the Mg–CO_2_–H_2_O system and the thermal decomposition of dypingite, Mg_5_(CO_3_)_4_(OH)_2_·5H_2_O: Implications for geosequestration of carbon dioxide. Chem. Geol..

[10] Kataki S., West H., Clarke M., Baruah D.C. (2016). Phosphorus recovery as struvite: Recent concerns for use of seed, alternative Mg source, nitrogen conservation and fertilizer potential. Resour. Conserv. Recycl..

[11] Wang J., Burken J.G., Zhang X., Surampalli R. (2005). Engineered Struvite Precipitation: Impacts of Component-Ion Molar Ratios and pH. J. Environ. Eng..

[12] Sena M., Hicks A. (2018). Life cycle assessment review of struvite precipitation in wastewater treatment. Resour. Conserv. Recycl..

[13] Gong W., Li Y., Luo L., Luo X., Cheng X., Liang H. (2018). Application of Struvite-MAP Crystallization Reactor for Treating Cattle Manure Anaerobic Digested Slurry: Nitrogen and Phosphorus Recovery and Crystal Fertilizer Efficiency in Plant Trials. Int. J. Environ. Res. Public Health.

[14] Capdevielle A., Sýkorová E., Biscans B., Béline F., Daumer M.L. (2013). Optimization of struvite precipitation in synthetic biologically treated swine wastewater-Determination of the optimal process parameters. J. Hazard. Mater..

[15] Daneshgar S., Buttafava A., Capsoni D., Callegari A., Capodaglio A.G. (2018). Impact of pH and ionic molar ratios on phosphorous forms precipitation and recovery from different wastewater sludges. Resources.

[16] Numviyimana C., Warchoł J., Izydorczyk G., Baśladyńska S., Chojnacka K. (2020). Struvite production from dairy processing wastewater: Optimizing reaction conditions and effects of foreign ions through multi-response experimental models. J. Taiwan Inst. Chem. Eng..

[17] González-Morales C., Camargo-Valero M.A., Molina-Pérez F.J., Fernández B. (2019). Effect of the stirring speed on the struvite formation using the centrate from a WWTP. Rev. Fac. Ing. Univ. Antioq..

[18] Shalaby M.S., El-Rafie S., Hamzaoui A.H., M’nif A. (2015). Modeling and optimization of phosphate recovery from industrial wastewater and precipitation of solid fertilizer using experimental design methodology. Chem. Biochem. Eng. Q..

[19] (2004). EU Commission Implementing Decision (Eu) 2018/1147. Establ. Best Available Tech. Conclus. Waste Treat..

[20] Finkbeiner M., Inaba A., Tan R.B.H., Christiansen K., Klüppel H. (2006). Commentaries the New International Standards for Life Cycle Assessment: ISO 14040 and ISO 14044.

[21] Tan Z., Lal R., Wiebe K. (2005). Global Soil Nutrient Depletion and Yield Reduction. J. Sustain. Agric..

[22] Kinidi L., Tan I.A.W., Abdul Wahab N.B., Bin Tamrin K.F., Hipolito C.N., Salleh S.F. (2018). Recent Development in Ammonia Stripping Process for Industrial Wastewater Treatment. Int. J. Chem. Eng..

[23] Tang H., Xu X., Wang B., Lv C., Shi D. (2020). Removal of ammonium from swine waste water using synthesized zeolite from fly ash. Sustainability.

[24] Ban Z.S., Dave G. (2004). Laboratory studies on recovery of n and p from human urine through struvite crystallisation and zeolite adsorption. Env. Technol..

[25] Huang H., Xiao D., Pang R., Han C., Ding L. (2014). Simultaneous removal of nutrients from simulated swine wastewater by adsorption of modified zeolite combined with struvite crystallization. Chem. Eng. J..

[26] Holub M., Balintova M., Demcak S., Hurakova M. (2016). Characterization of Natural Zeolite and Determination Its Adsorption Properties. J. Civ. Eng. Environ. Arch..

[27] Wasielewski S., Rott E., Minke R., Steinmetz H. (2018). Evaluation of different clinoptilolite zeolites as adsorbent for ammonium removal from highly concentrated synthetic wastewater. Water.

[28] Kotoulas A., Agathou D., Triantaphyllidou I., Tatoulis T., Akratos C., Tekerlekopoulou A., Vayenas D. (2019). Zeolite as a Potential Medium for Ammonium Recovery and Second Cheese Whey Treatment. Water.

[29] An S.W., Jeong Y.C., Cho H.H., Park J.W. (2016). Adsorption of NH_4_^+^-N and *E. coli* onto Mg^2+^-modified zeolites. Environ. Earth Sci..

[30] Shaddel S., Grini T., Ucar S., Azrague K., Andreassen J.P., Østerhus S.W. (2020). Struvite crystallization by using raw seawater: Improving economics and environmental footprint while maintaining phosphorus recovery and product quality. Water Res..

[31] Vudagandla S., Siva Kumar N., Dharmendra V., Asif M., Balaram V., Zhengxu H., Zhen Z. (2017). Determination of Boron, Phosphorus, and Molybdenum Content in Biosludge Samples by Microwave Plasma Atomic Emission Spectrometry (MP-AES). Appl. Sci..

[32] Santos W.O. (2019). Acid Ammonium Citrate as P Extractor for Fertilizers of Varying Solubility. Rev. Bras. Ciênc. Solo.

[33] Thant Zin M.M., Kim D.J. (2019). Struvite production from food processing wastewater and incinerated sewage sludge ash as an alternative N and P source: Optimization of multiple resources recovery by response surface methodology. Process Saf. Environ. Prot..

[34] Zhang T., Ding L., Ren H., Guo Z., Tan J. (2010). Thermodynamic modeling of ferric phosphate precipitation for phosphorus removal and recovery from wastewater. J. Hazard. Mater..

[35] Christensen J., Bastien C. (2016). Introduction to General Optimization Principles and Methods. Nonlinear Optimization of Vehicle Safety Structures.

[36] Chua L.H.C., Tan S.B.K., Sim C.H., Kumar M. (2012). Treatment of baseflow from an urban catchment by a floating wetland system. Ecol. Eng..

[37] Meza J.C. (2010). Steepest descent. Wiley Interdiscip. Rev. Comput. Stat..

[38] Ramprasad C., Alekhya D., Bhishmitha C., Deepika C.S. (2020). Precipitation of struvite by sustainable waste materials and use as slow release fertilizer—A circular economy approach. IOP Conf. Ser. Mater. Sci. Eng..

[39] Himmelbauer M.L., Loiskandl W., Kastanek F. (2004). Estimating length, average diameter and surface area of roots using two different Image analyses systems. Plant Soil.

[40] Holub M., Balintova M., Pavlikova P., Palascakova L. (2013). Study of sorption properties of zeolite in acidic conditions in dependence on particle size. Chem. Eng. Trans..

[41] Wang S., Peng Y. (2010). Natural zeolites as effective adsorbents in water and wastewater treatment. Chem. Eng. J..

[42] Stolzenburg P., Capdevielle A., Teychené S., Biscans B. (2015). Struvite precipitation with MgO as a precursor: Application to wastewater treatment. Chem. Eng. Sci..

[43] Ronteltap M., Maurer M., Gujer W. (2007). Struvite precipitation thermodynamics in source-separated urine. Water Res..

[44] Ulex G.L. (1845). CLXIII. On struvite, a new mineral. Mem. Proc. Chem. Soc..

[45] Whitaker A., Jeffery J.W. (1970). The crystal structure of struvite, MgNH_4_ PO_4_ 6H_2_O. Acta Cryst. Sect. B.

[46] Guo W., Nazim H., Liang Z., Yang D. (2016). Magnesium deficiency in plants: An urgent problem. Crop J..

[47] El Diwani G., El Rafie S., El Ibiari N.N., El-Aila H.I. (2007). Recovery of ammonia nitrogen from industrial wastewater treatment as struvite slow releasing fertilizer. Desalination.

[48] Yetilmezsoy K., Kocak E., Akbin H.M., Özçimen D. (2020). Utilization of struvite recovered from high-strength ammonium-containing simulated wastewater as slow-release fertilizer and fire-retardant barrier. Environ. Technol..

[49] Szőllősi R., Molnár Á., Kondak S., Kolbert Z. (2020). Dual effect of nanomaterials on germination and seedling growth: Stimulation vs. phytotoxicity. Plants.

